# A Newly Designed Primer Revealed High Phylogenetic Diversity of *Endozoicomonas* in Coral Reefs

**DOI:** 10.1264/jsme2.ME18054

**Published:** 2018-05-12

**Authors:** Jia-Ho Shiu, Jiun-Yan Ding, Ching-Hung Tseng, Shueh-Ping Lou, Takuma Mezaki, Yu-Ting Wu, Hsiang-Iu Wang, Sen-Lin Tang

**Affiliations:** 1 Molecular and Biological Agricultural Sciences Program, Taiwan International Graduate Program, Academia Sinica Taipei Taiwan; 2 Biodiversity Research Center, Academia Sinica Taipei Taiwan; 3 Graduate Institute of Biotechnology, National Chung-Hsing University Taichung Taiwan; 4 Biotechnology Center, National Chung-Hsing University Taichung Taiwan; 5 Bioinformatics Program, Taiwan International Graduate Program, Academia Sinica Taipei Taiwan; 6 Institute of Biomedical Informatics, National Yang-Ming University Taipei Taiwan; 7 Biological Institute on Kuroshio, Kuroshio Biological Research Foundation Kochi Japan; 8 Department of Forestry, National Pingtung University of Science and Technology Pingtung Taiwan

**Keywords:** *Endozoicomonas*, coral microbe, diversity, 16S rRNA gene, pyrosequencing

## Abstract

*Endozoicomonas* bacteria are commonly regarded as having a potentially symbiotic relationship with their coral hosts. However, their diversity and phylogeny in samples collected from various sources remain unclear. Therefore, we designed an *Endozoicomonas*-specific primer paired with a bacterial universal primer to detect the 16S ribosomal RNA (rRNA) genes of this taxon and conducted an in-depth investigation of the *Endozoicomonas* community structure in reef-building corals. The primer had high specificity in the V3–V4 region (95.6%) and its sensitivity was high, particularly when *Endozoicomonas* was rare in samples (*e.g.*, in seawater, which had a higher alpha diversity of *Endozoicomonas* than corals). In coral samples, predominant V3–V4 ribotypes had greater divergence than predominant V1–V2 ribotypes, and were grouped into at least 9 novel clades in a phylogenetic tree, indicating *Endozoicomonas* had high phylogenetic diversity. Divergence within this genus was potentially higher than that among 7 outgroup genera based on the phylogenetic distances of partial 16S rDNA sequences, suggesting that the taxonomy of this genus needs to be revised. In conclusion, dominant *Endozoicomonas* populations had variable phylogenies; furthermore, the newly designed primers may be useful molecular tools for the reliable detection of the *Endozoicomonas* community in marine environments.

Coral-associated bacteria are a crucial component in coral holobionts and are commonly considered to affect coral health, disease, and nutrient supply (19, 45, 49, 50, 58). A highly diverse coral-associated bacterial community was prevalent in many surveys (5, 9, 47), resulting in difficulties with defining the core coral microbiota and elucidating their functions. However, a few microbes likely to be core symbionts of the holobiont were recently identified (1); these organisms may have important functions (28) and beneficial roles in the coral holobiont.

Bacteria of the genus *Endozoicomonas* are commonly suggested as core coral microbes, and are closely associated with their coral hosts (8, 12, 40). These bacteria were detected in their coral hosts before bleaching and showed resilience after bleaching, suggesting ecological associations between *Endozoicomonas* and coral health (6, 33). Moreover, these bacteria were rarely detected in parts affected by coral diseases, but were abundant in uninfected parts, supporting the assertion that *Endozoicomonas* is associated with healthy coral (20, 37).

The genus *Endozoicomonas* was first proposed by Kurahashi and Yokota in 2007 (30), whereas the genus *Spongiobacter* was initially identified from a marine sponge; however, this genus has not been validated. These two genera are phylogenetically mixed and not clarified (27, 61). *Endozoicomonas* are common microbial residents that are present not only in corals, but also in other marine invertebrates worldwide. They were recently reported in various marine invertebrates, *e.g.*, scleractinian corals (31), octocorals (10, 56), sponges (41), sea slugs (30), sea squirts (13), sea anemones (14), hydras (53), pen shells (25), polychaetes (43), oysters (61), and bivalves (27), and also in various locations, including South Africa (54), Asia (60), North America (22), Central America (37), South America (51), Europe (32), the Red Sea (4), and the Great Barrier Reef (35). Although the biogeographical prevalence of *Endozoicomonas* bacteria has been reported, their phylogeny and diversity remain unclear.

Variations in *Endozoicomonas* spp. have been reported and were suggested to be associated with habitats or host species (4, 34, 35, 42, 46). However, a comparative phylogenetic study using datasets from these studies is questionable due to variations in the methods used for the identification of these bacteria. Although the 16S rRNA gene was frequently used in previous studies, the various hypervariable regions of the 16S rRNA gene (*e.g.*, the V1–V3 or V4–V5 regions) used resulted in difficulties with comparing phylogenetic relationships, particularly at a lower taxonomic level. Moreover, 16S rRNA genes were commonly detected using a pair of universal primers for DNA amplification. The detection of *Endozoicomonas* may be unreliable, particularly when there is a limited bacterial population in samples. Hence, in order to categorize diversity and phylogenetic variations, a specific primer for the detection of *Endozoicomonas* is required.

In the present study, we designed a novel primer set that specifically targeted the 16S rRNA gene of *Endozoicomonas*, enabling detailed assessments of the *Endozoicomonas*-related community. Furthermore, the primers were tested with 30 samples from various sources (*i.e.*, 9 genera of reef-building corals, 3 locations, and 4 sampling times), and 3 seawater samples from 3 locations. Primer specificity and sensitivity were assessed and the alpha and beta diversities of *Endozoicomonas* were compared among samples. To the best of our knowledge, this is the first study to demonstrate the higher alpha diversity of *Endozoicomonas* in seawater versus coral samples, despite the markedly lower relative abundance of *Endozoicomonas* in seawater. Based on these results, in addition to the high phylogenetic diversity of *Endozoicomonas* among coral samples, *Endozoicomonas* may prefer a host-associated lifestyle and exhibit high heterogeneity in physiology, genetics, and ecology.

## Materials and Methods

### *Endozoicomonas*-specific primer design and pretest results

The *Endozoicomonas*-specific primer set was comprised of a specific reverse primer, En771R (5′-TCAGTGTCARRCCTGAGT GT-3′) and a bacterial universal forward primer, 27F (5′-AGAGTT TGATCMTGGCTCAG-3′). En771R was designed from the consensus region in the 16S rRNA genes of *Endozoicomonas* and *Spongiobacter* sequences published in the NCBI database (accession numbers: FJ457274.1, DQ889891.1, DQ917830.1, DQ917863.1, DQ917871.1, DQ917877.1, DQ917879.1, DQ917887.1, DQ917896.1, DQ917901.1, AB205011.1, AB196667.1, FJ347758.1, JX152780.1, and KC878324.1). The SINA Alignment service was used to confirm that these sequences were affiliated with *Endozoicomonas*. The selected consensus region differed from other close genera of *Hahellaceae* (accession numbers: EU599216.4, AY130994.1, AB467279.1, and AB467280.1). In addition, we designed a mismatch base (T) for the primer to be specific for *Endozoicomonas* based on a pre-test.

In the pretest, the mismatched primer (En771R: 5′-TCAGTGT CARRCCTGAGTGT-3′) and matched 771R primer (5′-TCAGTG TCARRCCAGAGTGT-3′) were compared using PCR and electrophoresis. We tested primer specificity and searched for a suitable annealing temperature by pairing our primer with the bacterial universal forward primer 27F in gradient PCR (range, 52.9 to 60.2°C). The V1–V4 region of the 16S rRNA genes of *E. elysicola*, *E. montiporae*, and *Simiduia agarivorans* and the total DNA of coral *Heliopora* samples were amplified. It is noteworthy that *S. agarivorans* is one of the groups closest to *Endozoicomonas*, and not classified as an *Endozoicomonas* spp. Furthermore, *S. agarivorans* was selected for the PCR pretest because 2 uncultivable bacteria that belonged to *Simiduia* from environmental samples were matched to a matched 771R primer when the primer pair was tested *in silico* using a SILVA primer test tool. Therefore, we used it as a negative control to select the annealing temperature.

A suitable annealing temperature for the primer pair was selected by gradient PCR (temperature range, 49.8 to 60.8°C) with finer temperature intervals (1–2°C). The V1–V4 regions of the 16S rRNA genes of *E. elysicola*, *E. montiporae*, *S. agarivorans*, and *Escherichia coli* (the latter 2 species were used as negative controls) were amplified with the specific primer En771R and bacterial universal primer 27F. In gel electrophoresis, 5 μL of each PCR product was loaded on a 1.5% agarose gel, and the gel was run in 1× TAE buffer under 100 V for 1 h. The specified regions in the 16S rRNA genes of *E. elysicola* and *E. montiporae* were amplified when the annealing temperature exceeded 54°C, whereas there were no PCR products from *S. agarivorans* or *E. coli* DNA. Therefore, PCR conditions were an initial step of 94°C for 3 min, 35 cycles of 94°C for 30 s, 54°C for 30 s and 72°C for 45 s, with a final extension of 72°C for 10 min.

### Sample collection

Permits for coral sampling were received from local governments. In order to detect *Endozoicomonas* in various reef-building coral samples, 30 healthy coral samples were collected from 3 locations (Table S1 and Fig. S1): Kenting (Taiwan, tropical region, 21°56′58.3″N, 120°45′11.9″E), Hemei (Taiwan, subtropical region, 25°05′34.45″N, 121°55′2.06″E), and Kochi (Japan, near temperate region, 32°46′42.95″N, 132°43′56.06″E). The taxonomic affiliations of the collected coral samples belonged to 9 genera and consisted of a robust clade in Hexacorallia (*Stylophora* and *Favia*), a complex clade in Hexacorallia (*Porites*, *Euphyllia*, *Acropora*, *Isopora*, and *Montipora*), Octocorallia (*Heliopora*), Zoantharia (*Palythoa*), and Hydrozoa (*Millepora*).

All corals and seawater were collected at depths of 5 to 7 m. Coral samples were collected using either bone scissors or a hammer and chisel, rinsed with sterilized seawater, and placed in 99% ethanol for transportation. Duplicate coral sampling was performed on 2 separate colonies of each selected coral genus, owing to limited financial support and sampling times. At each sampling site, 1 L of seawater was collected just before coral sampling, and then filtered through a cellulose acetate membrane with 0.2-μm pores (Advantec, Tokyo, Japan). Sample preparation, bench experiments, and bioinformatic analyses were summarized in a flowchart (Fig. S2).

### Total DNA extraction and amplification of the mitochondrial COI gene in coral

Coral samples were stored in 99% ethanol and washed twice with TE buffer (10 mM Tris-HCl and 1 mM EDTA, pH 8) before DNA extraction. Coral tissue with a skeleton was frozen in liquid nitrogen and then ground using a sterile mortar and pestle. The powder was transferred into TE buffer for total DNA extraction, as described (23, 59).

The taxonomy of coral samples (genus level) was assessed by morphology, and verified using DNA barcoding in a mitochondrial cytochrome c oxidase subunit I (COI) gene. The COI primers used in PCR for coral host identification were LCO1490 (5′-GGTCAA CAAATCATAAAGATATTGG-3′) and HCO2198 (5′-TAAACTT CAGGGTGACCAAAAAATCA-3′) (17). The 50-μL PCR mixture contained 1.5 U *TaKaRa Ex Taq* (Takara Bio, Otsu, Japan), 1× *TaKaRa Ex Taq* buffer, 0.2 mM deoxynucleotide triphosphate mixture (dNTP), 0.2 mM of each primer, and 40 ng DNA. The thermocycler conditions employed were an initial step of 94°C for 5 min, 30 cycles of 94°C for 30 s, 45°C for 20 s, and 72°C for 40 s, with a final extension of 72°C for 10 min (24). PCR products (~650 bp) were sequenced by Sanger sequencing; sequences identified by BLAST (NCBI) are shown (Table S2 and S1 Text).

### Amplification of the *Endozoicomonas* V1–V4 region in the 16S rRNA gene using specific primers, multiplex Roche 454 junior pyrosequencing, and sequence data processing

The V1–V4 regions of 16S rRNA genes in *Endozoicomonas* species were amplified with the specific primer En771R and bacterial universal primer 27F. PCR conditions were optimized in order to increase specificity and sensitivity to the target gene. PCR occurred in 50-μL reaction volumes, consisting of 2.5 U *TaKaRa Ex Taq*, 1× *TaKaRa Ex Taq* buffer, 0.2 mM dNTP, 0.25 mM of each primer, and 50 to 150 ng purified total DNA. The thermocycler was set to an initial step of 94°C for 3 min, 35 cycles of 94°C for 30 s, 54°C for 30 s and 72°C for 45 s, and a final extension of 72°C for 10 min. In order to tag each PCR product (~750 bp) with a unique barcode sequence, each tagged primer was designed with 4 overhanging nucleotides at the 5′ ends of the common primers En771R and 27F. The adding reaction was performed with 5-cycle PCR with modified primers; each cycle was run at 94°C for 30 s, 55.7°C for 20 s, and 72°C for 45 s. In Acr2S1 and Acr3S2 samples, due to the lower yield of PCR products, 40 cycles were used at Stage 2 of PCR amplification, and 10 cycles at Stage 2 of tagging PCR.

Final tagging PCR products were pooled and sequenced in 2 independent Roche 454 GS junior with Titanium chemistry pyrosequencing runs at the Institute of Molecular Biology, Academia Sinica (Taipei, Taiwan). Overall, 404,337 raw reads were obtained from pyrosequencing. Reads with any ambiguous bases (N) or <380 bp were excluded. According to specific barcode sequences corresponding to each sample, the remaining reads were sorted using our in-house pipeline (http://140.109.29.21/trimmer/; http://140.109.29.21/sorter/) (9). Potential chimeras were identified by UCHIME (15) and Chimera Slayer (21) and removed. A total of 241,539 qualified sequencing reads from both ends of the amplicons of the 16S rRNA gene were obtained after quality trimming and chimera checking.

### Amplification of the bacterial V1–V2 region using the bacterial universal primer, pyrosequencing, and data processing

In 8 coral *Acropora* samples and 1 seawater sample collected from Kochi, regions V1–V2 of the bacterial 16S rRNA gene were amplified by PCR using the bacterial universal primers 27F and 341R (5′-CTGCTGCCTCCCGTAGG-3′). DNA tagging PCR was used to fuse unique tags to each PCR product, which was conducted as described (2). Amplicons from the 9 Kochi samples were quantified and pooled in equal amounts. Multiplex sequencing was performed with a Roche 454 GS junior with Titanium chemistry - System (Roche 454 Life Sciences, Branford, CT, USA) at Mission Biotech (Taipei, Taiwan).

Raw sequencing reads were sorted into samples according to barcodes using an in-house sorter script (http://140.109.29.21/scripts/). After sorting and trimming with data from specific primers, high-quality reads were extracted using MOTHUR (52) with the following criteria: 1) read lengths between 280 and 350 bp; 2) average quality score >20; 3) homopolymer length <8 bp; and 4) removal of reads with any ambiguous base (N). Thereafter, the 4 nucleotide tags and primer sequences were removed. Chimeric reads were inspected and eliminated by UCHIME (15) with USEARCH v7.0.1090 (parameters: reference mode, rdp_gold database, and mindiv of 3). A total of 91,211 qualified sequences were retained for subsequent analyses.

### Taxonomic identification of V1–V2 and V3–V4 region sequences for primer specificity

A total of 165,481 V1–V2 and 76,058 V3–V4 qualified sequences were obtained using the specific primer in PCR, whereas there were 91,211 qualified sequences obtained using the bacterial universal primer. Their taxa were identified using the Naïve Bayesian Classifier (v16) (57) of the Ribosomal Database Project (RDP, release no. 10.31), with a bootstrap value of 0.8. A bar chart of the bacterial composition from the specific primer was presented after discarding 276 and 507 unclassified sequences (No hit) in the V1–V2 and V3–V4 sequences, respectively.

In order to assess the specificity of our designed primer, unclassified sequences of *Bacteria*, *Proteobacteria*, *Gammaproteobacteria*, *Oceanospirillales*, and *Hahellaceae* in the histogram were further classified by searching against the SILVA and Greengenes databases (v128 and ver. gg_13_8_99) implemented in MOTHUR. Sequences annotated as *Endozoicomonas* in the RDP database or *Endozoicimonaceae* in the Greengenes database were selected for subsequent analyses.

### Unique sequence profile, diversity index estimation, and rarefaction curve construction

The 13,693 V1–V2 and 3,633 V3–V4 distantly related *Endozoicomonas* sequences, defined by the Classifier of RDP (v16) and Greengenes database (gg_13_8_99) (36), were discarded. The remaining 151,788 V1–V2 and 72,425 V3–V4 sequences were subjected to further analyses.

In order to compare compositional variations in *Endozoicomonas* among samples on a finer scale, all remaining V1–V2 and V3–V4 sequencing reads affiliated with *Endozoicomonas* were aligned against the 28 aligned *Endozoicomonas*-related sequences downloaded from NCBI (accession numbers of sequences: HE818335.1, HE818343.1, AB695089.1, DQ917901.1, FJ347758.1, JX488685.1, JX488684.2, AB196667.1, JX152780.1, KC878324.1, DQ889929.1, DQ917830.1, DQ889931.1, DQ889906.1, DQ889891.1, DQ889911.1, DQ917896.1, DQ917887.1, FJ457274.1, AB205011.1, DQ917871.1, DQ917863.1, DQ917879.1, DQ917877.1 EU599216.4, AY130994.1, AB467280.1, and AB467279.1) using Nearest Alignment Space Termination (NAST) (11). Two V3–V4 reads in the alignment that lacked homologous sequences were removed. The qualified alignment was trimmed to a consistent length. A total of 51,934 V1–V2 and 28,349 V3–V4 unique reads were obtained after removing redundant identical sequences. The ratio of each unique sequence in each sample was calculated and profiled using R language (www.r-project.org). The ratio of each unique sequence in each sample was calculated by the read number of each unique sequence, divided by the total read number in each sample, and values were then transformed to a percentile.

Qualified multiple sequence alignments were used in subsequent analyses using the UPARSE pipeline (16), including the assessment of operational taxonomic units (OTUs) with a cut-off at 3% divergence at a species-like level, the composition of OTUs in each sample, and the estimation of indices for alpha diversity (*i.e.*, values of richness, Shannon-Weaver index, Gini-Simpson index, and evenness). In UPARSE, de-replication was performed and singleton was included (options: –derep_prefix and –minsize 1). Alpha diversity was also present after rarefying to an even 500 sequence depth in each sample by USEARCH (v9.2; options: -otutab_norm).

### Phylogenetic analysis of 16S rRNA gene sequences of *Endozoicomonas* and *Spongiobacter*

**Fig. 1:**
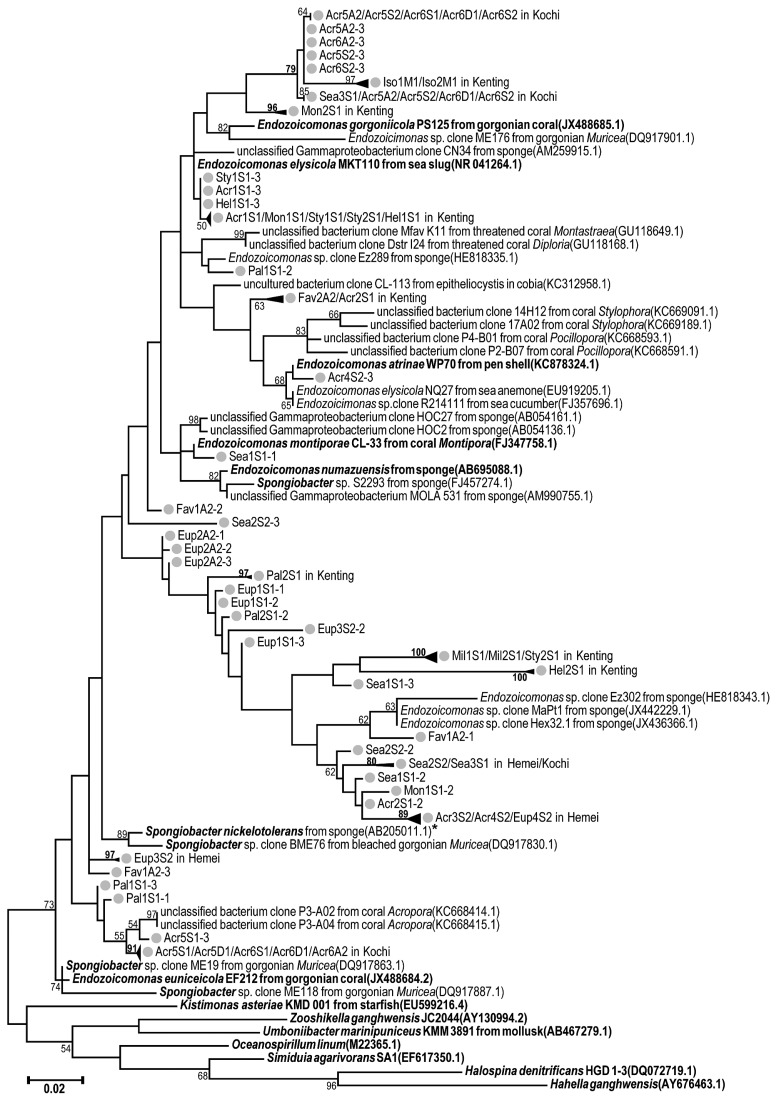
Phylogenetic tree of *Endozoicomonas* and *Spongiobacter* 16S rRNA sequences and closely related bacteria. Phylogeny was constructed with the top 3 abundant unique sequences of the V3–V4 region in the 16S ribosomal RNA gene in each sample, and other members of *Endozoicomonas*, *Spongiobacter*, and representative sequences in the family, *Hahellaceae*, the order, *Oceanospirillales*, and the phylum, *Gammaproteobacteria*, in GenBank. Sequences collected from this study were marked as grey circles. Numbers shown on branches are bootstrap values (1,000 bootstraps; those <50% are not shown). The scale bar corresponds to 0.02 substitutions per nucleotide position. The bold font denotes *Spongiobacter* or isolated strains of *Endozoicomonas*, and the first *Spongiobacter* sequence proposed in 2005 was marked with an asterisk.

In the analysis of the phylogenetic relationships of unique *Endozoicomonas* sequences detected in samples, the phylogenetic tree included the top 3 most abundant unique sequences from each sample, and 31 reference sequences of the 16S rRNA genes of *Spongiobacter* and *Endozoicomonas* (downloaded from GenBank; accession numbers are listed in Fig. 1) were included. In addition, 7 reference sequences as outgroups in the phylogenetic tree downloaded from GenBank belonged to the family *Hahellaceae* (*i.e.*, *Kistimonas asteriae*, *Zooshikella ganghwensis*, *Halospina denitrificans*, and *Hahella ganghwensis*), the order *Oceanospirillales* (*i.e.*, *Oceanospirillum linum*), and unclassified *Gamma-proteobacteria* (*i.e.*, *Umboniibacter marinipuniceus* and *S. agarivorans*), which are phylogenetically close to *Endozoicomonas* (48, 60).

The tree, based on the V1–V2 or V3–V4 datasets, was generated using the maximum-likelihood method with the Tamura-Nei model and 1,000 bootstrap replicates in MEGA 7 (29). All base positions containing gaps or missing data in the sequence alignment were discarded. A total of 305/415 informative sites in the alignment of V1–V2/V3–V4 data were available for the analysis in the phylogenetic tree.

Divergence among all 137 sequences in the tree was assessed by pairwise distance estimates of evolutionary divergence with a Kimura 2-parameter model in MEGA 7 (29), and performed as a boxplot using the R platform.

In order to examine the uncertain relationship between *Endozoicomonas* and *Spongiobacter*, 28 of the nearly full-length 16S rRNA gene sequences of both bacterial groups were downloaded from GenBank as representative sequences for a relation analysis (accession numbers of sequences: HE818335.1, HE818343.1, AB695089.1, DQ917901.1, FJ347758.1, JX488685.1, JX488684.2, AB196667.1, JX152780.1, KC878324.1 for *Endozoicomonas* group; DQ889929.1, DQ917830.1, DQ889931.1, DQ889906.1, DQ889891.1, DQ889911.1, DQ917896.1, DQ917887.1, FJ457274.1, AB205011.1, DQ917871.1, DQ917863.1, DQ917879.1, and DQ917877.1 for the *Spongiobacter* group; and EU599216.4, AY130994.1, AB467280.1, and AB467279.1 for the outgroup). The divergence of selected sequences was assessed by distance estimates of average evolutionary divergence with the Kimura 2-parameter model in MEGA 7. All base positions containing gaps or missing data in the sequence alignment were discarded; thereafter, 1,151 informative sites in the alignment were available for analysis.

### Identification of V1–V2 amplicons from universal or specific primers for primer sensitivity

In order to assess the sensitivity of the *Endozoicomonas*-specific primer, the V1–V2 amplicon dataset from using specific primers in PCR was compared to the other V1–V2 amplicon dataset from using bacterial universal primers. In order to compare alpha diversity and the community composition of *Endozoicomonas* spp. in both datasets, *Endozoicomonas* sequences in the 9 Kochi samples from both datasets were combined into a single file, aligned using MOTHUR (52), and then assigned OTUs with a cut-off value at 97% identity by the UPARSE pipeline (16), as described above.

The alpha diversity and composition of *Endozoicomonas* OTUs in each Kochi sample were compared after rarefying to an even 2,496 sequence depth in each coral sample, based on the least number of sequences among Kochi coral samples, except the seawater sample in Kochi. After excluding singleton OTUs, the OTU composition in each Kochi sample from both datasets was presented in a bar chart, and the total number of *Endozoicomonas* OTUs in each dataset was also calculated.

### Data accessibility

Multiplex sequenced reads (the bacterial 16S region) were deposited in the NCBI Sequence Read Archive under BioProject (PRJNA268432). Accession number: SUB755584 for V3–V4 sequences amplified by specific primers, SUB2807188 for V1–V2 sequences amplified by specific primers, and SUB2990890 for V1–V2 sequences amplified by universal primers.

## Results

### Specificity of matched and mismatched primers for detecting

**Fig. 2:**
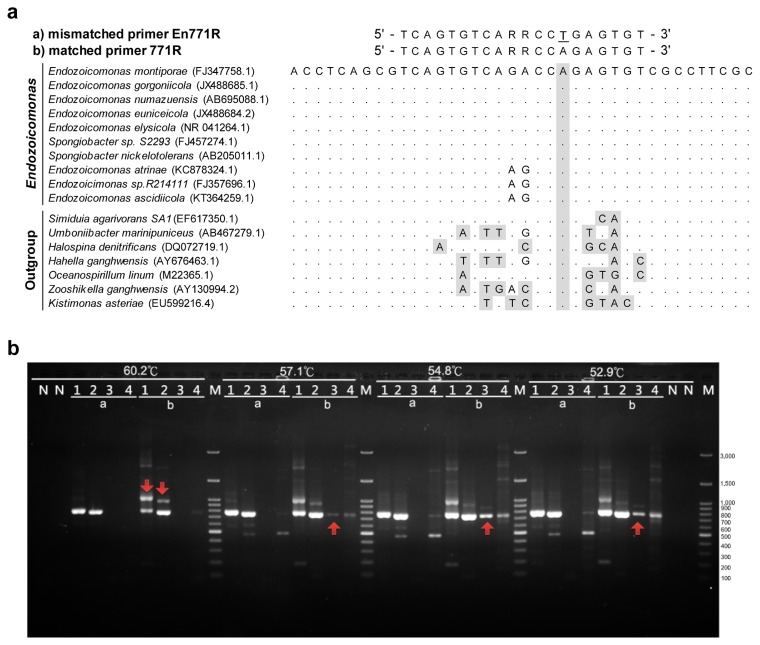
*Endozoicomonas*-specific primer design and test. (a) In primer design, a multiple sequence alignment includes mismatched En771R (marked as “a”), matched 771R primers (marked as “b”), 10 representative *Endozoicomonas* and *Spongiobacter* sequences, and 7 closely related outgroup sequences. The following IUPAC ambiguity codes are used in primers: R=A/G. One base T mismatched was designed and marked as the bottom line in the mismatched primer En771R. Dots indicate sequence identities to the reference sequence. Mismatched positions to the En771R primer are highlighted in grey. (b) Agarose gel electrophoresis of annealing temperature gradient PCR was used for the primer test. The V1–V4 region of 16S rRNA genes in *E. montiporae* (lane 1), *E. elysicola* (lane 2), and *S. agarivorans* (lane 3) and the total DNA of coral *Heliopora* samples (lane 4) were amplified in annealing temperature gradient (52.9, 54.8, 57.1, and 60.2°C) PCR with primer pairs, the bacterial universal primer 27F, and mismatched En771R (a) or no-mismatched 771R (b). “N” is a non-template control in PCR using the same primer pairs with annealing temperatures, 52.9 or 60.2°C. “M” is the DNA ladder marker. The expected size of PCR products is ~750 bp. Red arrows indicate non-specific products amplified using the matched 771R (b) and bacterial universal 27F primer pair.

Endozoicomonas The designed *Endozoicomonas-*specific reverse primer (771R) with 1 base mismatched (En771R: 5′-TCAGTGTC ARRCCTGAGTGT-3′) was compared to the reverse primer designed without mismatching (matched 771R: 5′-TCAGT GTCARRCCAGAGTGT-3′) by both being paired with the bacterial universal forward primer 27F in PCR (Fig. 2). We designed a one-base mismatched primer (En771R) to increase variations between the primer and sequences of *S. agarivorans* because there are 3 CAG repeats in the matched 771R primer and 4 CAG repeats in the region of *S. agarivorans* (Fig. 2a). We tested primer specificity and searched for a suitable annealing temperature by gradient PCR (range: 52.9 to 60.2°C).

Using the mismatched En771R primer, the specified region (~750 bp) in the 16S rRNA genes of *E. elysicola* and *E. montiporae* was amplified when the annealing temperature was 52.9–60.2°C, whereas there were no PCR products from cultivable *S. agarivorans* DNA (Fig. 2b). However, the 16S rRNA gene of *S. agarivorans* was amplified with the matched 771R primer under annealing temperatures from 52.9 to 57.1°C (red arrow in Fig. 2b). When the annealing temperature was increased to 60.2°C, there was no non-specific PCR product from *S. agarivorans* DNA using the matched primer or mismatched primer. However, the condition also yielded more non-specific 1-kb products (red arrow in Fig. 2b) from *E. montiporae* and *E. elysicola* DNA and a weak target band from coral *Heliopora* DNA, which had abundant *E. elysicola* close-relatives. Therefore, the mismatched primer (En771R) was selected for the subsequent primer test.

After the mismatched En771R was considered suitable for the detection of *Endozoicomonas* in subsequent experiments, we also paired this reverse primer with other forward primers (*i.e.*, bacterial universal 341F or *Endozoicomonas*-specific forward primers that we designed) in order to amplify a shorter PCR product (<400 bp) for Roche 454 GS junior pyrosequencing in 2013. However, none of these combinations had better specificity and yield than the 27F/En771R primer pair (tests were performed with gradient PCR and electrophoresis; data not shown).

### Specificity of designed primers for the detection of *Endozoicomonas* 16S rRNA genes

In order to examine the specificity and sensitivity of the primer pair 27F/En771R for detecting *Endozoicomonas* in coral samples, 30 coral samples that belonged to 9 genera and 3 seawater samples were collected from 3 locations (Table S1). The V1–V4 regions of 16S rRNA genes in *Endozoicomonas* species were amplified with the primer pair and amplified products were sequenced in a Roche 454 GS junior pyrosequencing. The qualified forward and reverse reads (380–550 bp) from pyrosequencing were shorter than the PCR products (~750 bp); therefore, we separately analyzed the forward and reverse reads (datasets for the V1–V2 and V3–V4 regions, respectively).

**Fig. 3:**
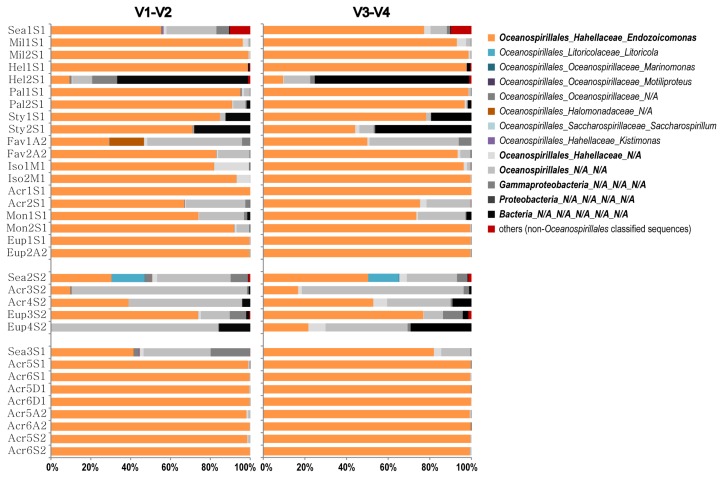
Bar chart of bacterial composition. Bacterial genera under the order *Oceanospirillales* are displayed, whereas the other classified non-*Oceanospirillales* taxa are grouped in the category “others” (red). All samples had sequences taxonomically assigned to *Endozoicomonas* (orange), and some samples had many unclassified sequences (grayscale). Unclassified sequences were selected and further classified against the SILVA and Greengenes databases. Abbreviations: Iso: *Isopora*; Sty: *Stylophora*; Eup: *Euphyllia*; Mon: *Montipora*; Acr: *Acropora*; Pal: *Palythoa*; Hel: *Heliopora*; Mil: *Millepora*; Fav: *Favia*; and Sea: seawater. Sampling times: M1: March 2011; S1: September 2011; D1: December 2011; A2: April 2012; and S2: September 2012.

In order to estimate specificity, each qualified bacterial sequence in both datasets was classified. The V3–V4 region of 16S rRNA tag sequencing yielded 86.4% sequences (65,305/75,551 sequences) affiliated to the genus *Endozoicomonas* by the method Classifier of RDP (v16). Every sample had *Endozoicomonas*, and most were comprised of >80% *Endozoicomonas* sequences (Fig. 3). Notably, some coral samples had many unclassified sequences (grayscale bars and bold keys in Fig. 3), particularly samples from Hemei, Taiwan. A total of 13.1% of V3–V4 sequences (9,895/75,551 sequences) were assigned as unclassified *Hahellaceae*, *Oceanospirillales*, *Gammaproteobacteria*, *Proteobacteria*, or *Bacteria* in RDP.

Among the 9,895 unclassified sequences in all samples, approximately 59.5% (5,884/9,895 sequences) or 70.2% (6,948/9,895 sequences) were assigned to *Endozoicomonas* in the SILVA database (v.128) or to *Endozoicimonaceae* in the Greengene database (ver. gg_13_8_99). A total of 86.4 to 95.6% (65,305 plus 6,948 in all 75,551 sequences) of the qualified sequences among samples corresponded to *Endozoicomonas*-related species in the V3–V4 region dataset.

In the V1–V2 region dataset, 81.3% sequences (134,325/165,205 sequences) affiliated to the genus *Endozoicomonas* with the method Classifier of RDP v16 (Fig. 3), and there were 17.4% unclassified *Hahellacea*, *Oceanospirillales*, *Gammaproteobacteria*, *Proteobacteria*, and *Bacteria* sequences (28,813) detected in all samples. We further classified these 28,813 unclassified sequences against the SILVA or Greengenes database (v.128 and ver. gg_13_8_99); using various databases, approximately 38.7% (11,157/28,813) or 60.6% (17,463/28,813 sequences) were identified as *Endozoicomonas* or *Endozoicimonaceae*, respectively.

Collectively, approximately 91.9% (134,325 plus 17,463 in all 165,205 sequences; 151,788/165,205) of the qualified V1–V2 sequences and 95.6% (72,253/75,551 sequences) of the qualified V3–V4 sequences corresponded to *Endozoicomonas-*related species.

### Sensitivity of designed primers for the detection of *Endozoicomonas*

**Fig. 4:**
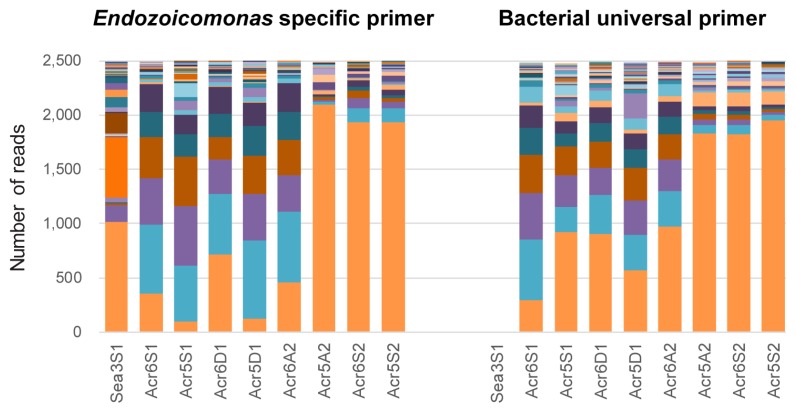
Bar chart of *Endozoicomonas* OTU compositions in coral and seawater samples collected from Kochi. Different colors present different OTUs in each dataset. The left panel shows the dataset amplified using *Endozoicomonas*-specific reverse primers paired with bacterial universal forward primers (27F and En771R), and the right panel shows the dataset from bacterial universal primer pairs (27F and 341R). The X axis lists the sample names. The Y axis is the number of reads in OTUs or samples after rarefying to 2,496 sequences per sample, except the seawater sample Sea3S1 from the bacterial universal primer, which had only 1 sequence belonging to *Endozoicomonas*. After rarefying and excluding singleton OTUs, there were 138 and 103 *Endozoicomonas* OTUs in the specific primer and universal primer datasets, respectively. This bar chart with color keys of all OTUs is shown in Fig. S5. Abbreviations in sample names: coral samples *Acropora* (Acr) and seawater samples (Sea); for sampling times, September 2011 (S1), December 2011 (D1), April 2012 (A2), September 2012 (S2).

In order to evaluate the sensitivity of the specific primer En771R, high variable V1–V2 sequences amplified from *Endozoicomonas*-specific primers (27F and En771R) were compared to V1–V2 sequences amplified from bacterial universal primers (27F and 341R). The V1–V2 sequences assigned as *Endozoicomonas* in 9 Kochi samples were selected for comparison. The number of sequences in each sample was rarefied to an even 2,496 sequence depth in each sample, which is the least number of sequences among all Kochi samples, except the seawater sample, in a universal primer dataset, and singleton OTUs were removed to prevent the overestimation of sensitivity. There were 159 *Endozoicomonas* OTUs, and the specific primer dataset had 138 *Endozoicomonas* OTUs, more than 103 *Endozoicomonas* OTUs in the universal primer data (Fig. 4 and Table 1). They shared 82 common OTUs between 2 datasets.

Based on common abundant OTUs, OTU compositions in samples were similar between primer sets (Fig. 4), as were the alpha diversities, estimated including singleton OTUs, of each sample between 2 datasets (Table 1). Notably, only 1 sequence in seawater was classified as *Endozoicomonas* when using the universal primer. In contrast, when using the specific primer, there were more *Endozoicomonas* sequences, with 88 OTUs detected in the seawater sample (that included singleton OTUs after rarefying), even more than those in coral samples.

### Diversity of the *Endozoicomonas* community in corals and seawater categorized with specific primers

In order to use our newly designed specific primer En771R paired with the 27F primer to characterize the composition of the *Endozoicomonas* community in corals from various sources, the alpha and beta diversities of *Endozoicomonas* in coral and seawater samples were estimated based on V1–V2 and V3–V4 sequences in the specific primer dataset. The alpha diversity, including the Shannon index, Gini-Simpson index, and evenness, of coral samples was lower than that of seawater samples (Table 2). For example, the Shannon index of *Endozoicomonas* was higher in seawater samples (2.34 to 3.31), but lower in coral samples (range 0.08 to 2.61, with most <2.0). In coral, diversities were also variable among samples. For example, samples of *Favia* (Fav1A1 and Fav1A2) and *Palythoa* (PalS1 and PalS2) from Kenting had more OTUs than others, whereas *Millepora* samples (Mil1S1 and Mil2S1) from Kenting had lower values.

Since there were only a few samples with a lower number of sequences (<500), diversity indices were rarefied to a 500 sequence depth (Table S3). Although the number of OTUs and values of richness, evenness, and diversity decreased after rarefying, comparative results of these values did not change among samples.

**Fig. 5:**
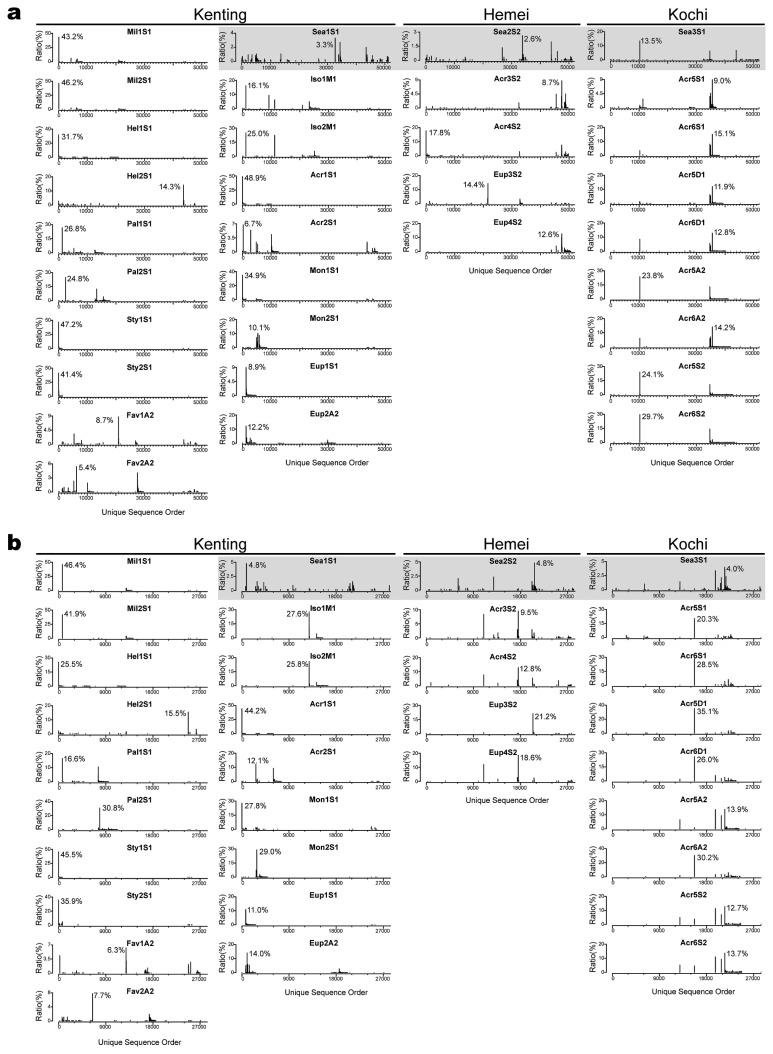
Profile of unique V1–V2 and V3–V4 sequences of *Endozoicomonas* in each sample. This profile illustrates the dominant *Endozoicomonas* group in coral and seawater samples (grey background) from (a) the V1–V2 region and (b) V3–V4 region of 16S rRNA datasets. The Y axis is the ratio of each unique group in each sample. The highest ratios of unique sequences in each sample are marked beside the sequences.

In order to detect the beta diversity of *Endozoicomonas* at the subtype level and prevent overlooking finer scale variations among samples, the unique sequence profiles of *Endozoicomonas* were analyzed (Fig. 5). Each unique sequence was comprised of identical sequences only as a proxy for a subtype. In the V3–V4 dataset (Fig. 5b), the proportions of the most abundant unique sequences in each coral sample ranged between 6.3 and 46.4%, whereas these proportions were all <5.0% in the 3 seawater samples. There was a similar pattern in the V1–V2 dataset (Fig. 5a), except for seawater samples from Kochi (Sea3S1).

Furthermore, the *Endozoicomonas*-subtype signatures of coral samples differed from those of seawater samples derived from the same location. Most of the profiles of 2 biological repeats of coral samples were similar and had the same highest unique sequences. Variations among coral samples were generally discernible, and resulted from differences among samples for the most abundant subtypes.

### Phylogenetic distance of *Endozoicomonas* and its related species when using the specific primers

In order to comprehend the phylogenetic analysis of the dominant 16S rRNA gene sequences of *Endozoicomonas*, the top 3 abundant unique sequences from each sample and 31 reference sequences of the 16S rRNA gene of *Spongiobacter* and *Endozoicomonas* and 7 outgroup sequences (downloaded from GenBank; accession numbers are listed in Fig. 1) were included.

**Fig. 6:**
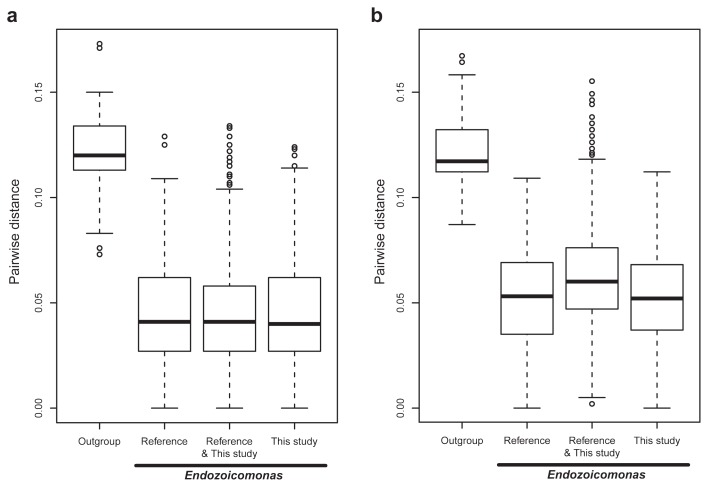
Boxplot of divergence among *Endozoicomonas* 16S rRNA sequences and closely related bacteria. Top 3 abundant *Endozoicomonas* unique sequences from (a) the V1–V2 region and (b) V3–V4 region of 16S rRNA in each sample in the present study, 31 reference sequences of the 16S rRNA genes of *Spongiobacter* and *Endozoicomonas* downloaded from GenBank, and representative sequences in the family, *Hahellaceae*, the order, *Oceanospirillales*, and the phylum, *Gamma-proteobacteria*, in GenBank (Outgroup) were assessed for divergence based on pairwise distances in the boxplot. Pairwise distances between sequences in this study and from GenBank were also performed (this study & reference). Divergence between all 137 sequences was assessed by distance estimates (Kimura 2-parameter model). Mean distances within or between groups was shown in Table S4.

*Endozoicomonas*-related sequences were highly diverse and demonstrated divergence, particularly in the V3–V4 region (Fig. 6). Pairwise distances among *Endozoicomonas*or *Spongiobacter*-related sequences ranged between 0 and 0.155 nucleotide substitutions per site in V3–V4 data (Fig. 6b), which were higher than those in V1–V2 data (from 0 to 0.134 nucleotide substitutions per site; Fig. 6a), whereas distances among 7 outgroup sequences, which belonged to the *Hahellaceae* family (*K. asteriae*, *Z. ganghwensis*, *H. denitrificans*, and *H. ganghwensis*), *Oceanospirillales* order (*O. linum*), and even unclassified *Gamma-proteobacteria* (*S. agarivorans* and *U. marinipuniceus*), ranged between 0.087 and only 0.167 nucleotide substitutions per site in V3–V4 data (Fig. 6b). All 3 medians of pairwise distances in the *Endozoicomonas* group were >0.05 nucleotide substitutions per site (at the genus level) in V3–V4 data (Fig. 6b), even though many pairwise distances were 0 nucleotide substitutions per site between sequences in the *Endozoicomonas* group. In contrast, the 3 medians of the pairwise distance in *Endozoicomonas* were <0.05 nucleotide substitutions per site in V1–V2 data (Fig. 6a). In V1–V2 and V3–V4 data, many values of pairwise distances in the *Endozoicomonas* group were >0.1 nucleotide substitutions per site, consistent with variations in the order level.

In order to estimate the phylogenetic diversity of *Endozoicomonas* in coral samples, the V3–V4 region had better resolution than the V1–V2 region for the phylogenetic analysis at the genus level (Fig. 1 and S4). The 7 outgroup sequences in the phylogenetic tree based on V1–V2 data were mixed with *Endozoicomonas* sequences (Fig. S4), whereas outgroup sequences were clearly separated from *Endozoicomonas* and *Spongiobacter* sequences in a phylogenetic tree constructed from the V3–V4 region (Fig. 1). Therefore, we selected only the V3–V4 region in subsequent phylogenetic analyses. Many *Endozoicomonas* species detected in the present study were novel clades from sequences in public databases (Fig. 1). For example, all of the top 3 abundant *Endozoicomonas* sequences from *Millepora* in Kenting had a high bootstrap value (100) as a new group in a phylogeny tree. In addition, there were at least 9 new *Endozoicomonas* clades, with a bootstrap value >70 (marked as bold in Fig. 1), detected by our newly designed primers.

Despite the unusually high variation within the *Endozoicomonas* group, including *Spongiobacter* (Fig. 6), *Spongiobacter* and *Endozoicomonas* were highly related in the phylogenetic tree (Fig. 1). The high bootstrap value (73) supported branching of the clade of *Endozoicomonas*/ *Spongiobacter* from the other outgroup genera, such as *Kistimonas* and *Zooshikella* (that belonged to the same family of *Hahellaceae*). In order to clarify the relationship between *Endozoicomonas* and *Spongiobacter*, 28 nearly full-length 16S rRNA genes (1,151 informative sites in total) of *Endozoicomonas* and *Spongiobacter* were compared using phylogenetic distances to estimate divergence among sequences. Average distances within the *Endozoicomonas* or *Spongiobacter* groups were 0.049 (*SE*=0.004) and 0.036 (*SE*=0.003), respectively. Similarly, the average distance between *Endozoicomonas* and *Spongiobacter* was 0.048 (*SE*=0.003). In contrast, average distances between *Endozoicomonas* or *Spongiobacter* and other members from *Hahellaceae* (*Kistimonas* and *Zooshikella*) or a closely related sequence (*Umboniibacter*) were markedly higher at 0.087 and 0.092 (*SEs*=0.006 and 0.007). Hence, the distance between *Endozoicomonas* and *Spongiobacter* was markedly smaller than other genera in the family *Hahellaceae*.

## Discussion

### The newly designed primer was highly specific for the detection of *Endozoicomonas*

We successfully designed what is apparently the first specific primer for the detection of *Endozoicomonas* for a community study and used it to measure changes in the *Endozoicomonas* community in corals and seawater (in which *Endozoicomonas* were very rare; Fig. S3) (3, 26). This primer was highly accurate in the V3–V4 region (95.6%) and specific for the detection of *Endozoicomonas*. The lower specificity in the V1–V2 region (91.9%) may have been due to less discrimination for identifying bacterial taxonomy at a genus level using the V1–V2 region than the V3–V4 region (Fig. 1 and S4), and the lack of an informative V1–V2 region in *Endozoicomonas* reference sequences in the database. Therefore, we recommend using the V3–4 region of specific primer-amplified 16S rDNA due to its high accuracy and specificity, making it more suitable for interpreting the community of *Endozoicomonas*.

### The newly designed primer was sensitive for the detection of *Endozoicomonas*

Primer sensitivity was assessed by comparisons with the universal primer in the V1–V2 region, and was also evident in the V3–V4 region based on the high divergence between the detected sequences of *Endozoicomonas* (Fig. 6) and new clades of *Endozoicomonas* identified (Fig. 1). In the V1–V2 datasets, specific primer data had similar numbers of *Endozoicomonas* OTUs with data from the universal primer in each sample (Fig. 4 and Table 1). Among all 9 samples, PCR with the specific primer detected more *Endozoicomonas* OTUs than that with the universal primer, but there were 21 *Endozoicomonas* OTUs detected only by the universal primer. The plausible reason for the undetectable by using the specific primer may result from the limited sequencing size from 2,496 to 3,330 sequences before rarefying (Table S5), but there are more than twice as the sequences (from 6,538 to 13,066) of samples detected using the universal primer. After rarefying all the samples down to 2,496 reads, those 21 OTUs detected only by the universal primer were represented by only 1 read for each OTU, which could be selected by chance and should be very low abundant in the samples.

However, more OTUs (56>21 OTUs) could be detected only by the specific primer after rarefying when the relative abundance of *Endozoicomonas* OTUs were low (Table 1, Fig. S5, and Sea3S1 sample in Fig. S3). In short, the sensitivity of the *Endozoicomonas*-specific primer for detecting *Endozoicomonas* was higher than that of the bacterial universal primer, especially for rare *Endozoicomonas* OTUs.

In analyses of the V3–V4 region, higher richness and diverse populations of *Endozoicomonas* in seawater than in coral sample were also detected (Table 2 and S3). *Endozoicomonas* in seawater was rare (3, 26) and barely detected using bacterial universal primers due to limitation of sequencing size and PCR bias. Furthermore, many *Endozoicomonas* species detected in the present study were novel clades (compared to sequences in public databases; Fig. 1). For example, the top 3 abundant *Endozoicomonas* sequences from *Millepora* in Kenting had a high bootstrap value as a new and monophyletic group in a phylogeny tree. In addition, our newly designed primers detected at least 9 new *Endozoicomonas* clades, with a bootstrap value >70. Moreover, sequences phylogenetically close to the representative cultivable species, *E. elysicola*, *E. montiporae*, *E. atrinae*, *E. gorgonicola*, and *E. eunicicola*, were all detected in our samples, suggesting a good detection range for the specific primer. Furthermore, the highly phylogenetic divergence of *Endozoicomonas* spp. was detected using our designed primer, in which the pairwise distances between *Endozoicomonas* sequences may be >0.1 nucleotide substitutions per site as the variation in order level (Fig. 6). The newly designed primer was very useful and has considerable potential to characterize the composition, diversity, dynamics, and location of *Endozoicomonas* in host cells.

### The V3–V4 region was superior to the V1–V2 region for a phylogenetic analysis of *Endozoicomonas*

The V1–V2 region had lower within-group distances of *Endozoicomonas* than the V3–V4 region (Fig. 6), whereas mean distances between *Endozoicomonas* and outgroup sequences were also lower in the V1–2 region than in the V3–V4 region (Table S4). This may account for the high bootstrap value, *i.e.*, 73, which supported a clade of *Endozoicomonas* and *Spongiobacter* away from other outgroup sequences in the V3–V4 region-based phylogenetic tree (Fig. 1), but not in the V1–V2 based phylogenetic tree (Fig. S4), despite higher divergence in the V3–V4 region than in the V1–V2 region from 16S rRNA in *Endozoicomonas*. In a previous study, the V3–V4 region was also reported to have better resolution for bacterial taxa identification, particularly at the genus level, and was also recommended for analyzing bacterial communities with either single-read or paired-end strategies (38). Therefore, we inferred that the V3–V4 region was superior to the V1–V2 region in 16S rRNA sequences for the phylogenetic analysis of *Endozoicomonas*.

### The host-associated lifestyle was more selective, but favorable for some *Endozoicomonas* sp

Our study appears to be the first to report a high level of *Endozoicomonas* diversity in seawater. The diversity of *Endozoicomonas* bacteria in corals was lower than that in seawater (Table 1 and S3), suggesting that environments in corals were more selective to *Endozoicomonas* than those in seawater. However, in previous studies, bacteria of the genus *Endozoicomonas* were identified at markedly higher relative abundance in corals than in seawater (3, 26) and the density of bacteria in corals was commonly 2.8- to 4.3-fold higher than that in seawater (18). Therefore, a part of *Endozoicomonas* bacteria may prefer to live in corals than in seawater.

Combining the results of the higher species diversity, but lower abundance of *Endozoicomonas* in seawater than in coral (Table 2 and Fig. 5), seawater from the nearby reef may have been a relatively neutral environment (compared to host association) for *Endozoicomonas*. In contrast, the host-associated environment for *Endozoicomonas* was more selective, but more favorable for partial populations of *Endozoicomonas*, in which the diversity of *Endozoicomonas* species was low and the relative abundance of dominant species increased in coral samples. Thus, our results also supported the hypothesis that *Endozoicomonas* preferred a host-associated lifestyle (12).

### Diverse relationships of *Endozoicomonas* and corals

The high variation among *Endozoicomonas*-related sequences in databases may have been due to differences in experimental procedures or strategies among studies. Nevertheless, variations in *Endozoicomonas* sequences from this study had an unusual level of diversity and divergence (Fig. 1 and 6). Based on the phylogenetic diversity and different host specificity of *Endozoicomonas* in the present study, we inferred that interactions between these bacteria and their coral hosts were diverse and complex. Previous studies indicated that bacteria were only present or abundant in healthy corals (6, 20, 37). However, other studies showed that *Endozoicomonas* bacteria were also dominant in unhealthy corals, such as those with white patch syndrome in *Porites* (54) and in water with eutrophication and overfishing (26). Based on these inventory studies, difficulties were associated with interpreting the role of these bacteria in corals. Nevertheless, based on a fluorescence *in situ* hybridization (FISH) analysis, *Endozoicomonas* bacteria gathered close to symbiotic algae in the endodermal tissues of *Stylophora pistillata* in the Red Sea, suggesting that these bacteria have specific habitats inside corals (4). Notably, some *Spongiobacter* bacteria were able to consume dimethylsulfoniopropionate (DMSP) produced by the symbiotic algae of corals (7, 44) that indirectly supported these bacteria detected close to the algae inside corals. However, many *Endozoicomonas* bacteria have been detected in the mucus layer of healthy corals (20, 33, 37, 39, 55), indicating that these bacteria have a broad habitat range with corals. Hence, the habitat variations of *Endozoicomonas* may be more profound than expected.

### A proposal to combine *Spongiobacter* and *Endozoicomonas* into a single taxon

Since the phylogenetic distance between the 2 genera (0.048 nucleotide substitutions per site) was smaller than that within the same genus (0.049 nucleotide substitutions per site), we propose to combine *Spongiobacter* and *Endozoicomonas* into a single taxon. The genus *Spongiobacter* was first proposed by Nishijima *et al.* in 2005 (unpublished), who described a nickel-tolerant bacterial isolate in a marine sponge. However, the genus name was not formally registered as a taxon. Nevertheless, with the publication of more marine invertebrate-associated microbial community surveys, several 16S rDNA sequences annotated as “*Spongiobacter* sp.” (based on the blast result in NCBI) were highly similar to *Endozoicomonas* species (61). Since the genus *Spongiobacter* lacked any specific description and no isolates were available in the authorized collection institutes, as well as a lack of evidence that distinguished this genus from *Endozoicomonas*, we proposed that *Spongiobacter* and *Endozoicomonas* be combined into the single genus *Endozoicomonas*. Furthermore, we may even consider *Endozoicimonaceae* or *Endozoicimonaceae* to be a new family (26, 13) due to the high divergence of this group (Fig. 1 and 6). In our view, unifying the nomenclature is critically important, particularly to facilitate phylogenetic diversity studies.

## Conclusions

Using a newly-designed specific primer, we detected the *Endozoicomonas* community in coral samples from various sources, different coral species, across divergent locations and at various times. The sensitivity and specificity of the specific primer were clearly evident. Many new *Endozoicomonas* 16S rDNA ribotypes and *Endozoicomonas* bacteria widely dispersed in all coral and seawater samples were detected. Based on lower diversity than seawater and only a few dominant ribotypes detected in each coral sample, we suggested that environments in corals were more selective to these bacteria than those in seawater. These dominant *Endozoicomonas* populations were highly variable; therefore, we infer that the *Endozoicomonas* community is highly diverse. Hence, searching for a consistent relationship between corals and *Endozoicomonas* appeared to be simplistic and perhaps inappropriate. Nevertheless, a comprehensive list of members in the genus *Endozoicomonas* will be important for identifying the co-occurrence of particular ribotypes of *Endozoicomonas* and coral species. Fortunately, we are optimistic that our new method will facilitate these studies. Our designed primer may also be used as a FISH probe to localize *Endozoicomonas* inside host cells.

## Supplementary Material



## Figures and Tables

**Table 1:** Sequence information and diversity estimates, including singleton OTUs after rarefying to an even 2,496 sequence depth, of the *Endozoicomonas* community in coral and seawater samples from Kochi, as represented in V1–V2 regions of the 16S rRNA gene, detected with bacterial universal primers (U) and *Endozoicomonas*-specific primers (S).

Sample	N^a^		OTU^b^			Richness^c^		Gini-Simpson		Shannon		Evenness^d^	
Primer	U	S	U	S	C	U	S	U	S	U	S	U	S
**Sea3S1**	1	2509	1	88	0	N.D.	0.416	N.D.	0.774	N.D.	2.20	N.D.	0.491
**Acr6S1**	2476	2500	53	69	29	0.407	0.643	0.863	0.843	2.28	2.16	0.575	0.509
**Acr5S1**	2474	2502	62	54	28	0.270	0.436	0.819	0.858	2.35	2.34	0.570	0.586
**Acr6D1**	2490	2498	71	56	37	0.514	0.509	0.816	0.827	2.27	2.12	0.531	0.526
**Acr5D1**	2480	2506	71	73	38	0.361	0.486	0.879	0.844	2.53	2.31	0.593	0.539
**Acr6A2**	2485	2500	57	60	36	0.362	0.492	0.796	0.841	2.12	2.14	0.525	0.523
**Acr5A2**	2480	2496	59	47	28	0.467	0.542	0.447	0.290	1.35	0.88	0.331	0.228
**Acr6S2**	2491	2500	53	58	31	0.370	0.492	0.460	0.397	1.37	1.14	0.346	0.282
**Acr5S2**	2484	2501	49	46	29	0.320	0.340	0.377	0.398	1.15	1.16	0.295	0.304

**Total**	–	–	103	138	82	–	–	–	–	–	–	–	–

U=Bacterial universal primers; S=*Endozocomonas*-specific primers; C=Common OTUs shared between U and S data.

N.D.=undetectable in samples.

a N defined as the number of sequences.

b Calculations were based on operational taxonomic units (OTUs) formed at an evolutionary distance of <0.03 (or approximately 97% similarity).

There were total 159 OTUs.

c Calculated as S/(N+1) where S is the number of singleton OTUs and N is the total number of OTUs.

d Shannon index divided by the logarithm of the number of OTUs

**Table 2:** Sequence information and diversity estimates using our newly designed *Endozoicomonas*-specific primers in V1–V2 and V3–V4 regions of the 16S rRNA gene.

Sample	N^a^		OTU^b^		Gini-Simpson		Shannon		Evenness^c^	
Primer	V1V2	V3V4	V1V2	V3V4	V1V2	V3V4	V1V2	V3V4	V1V2	V3V4
**Mil1S1**	5094	2132	21	11	0.11	0.03	0.26	0.10	0.08	0.04
**Mil2S1**	5626	2664	36	19	0.04	0.03	0.15	0.12	0.04	0.04
**Hel1S1**	**8829**	4336	56	27	0.10	0.04	0.31	0.14	0.08	0.04
**Hel2S1**	701	405	30	29	0.72	0.75	1.95	2.05	0.57	0.61
**Pal1S1**	4129	4917	81	47	0.85	0.72	2.43	1.56	0.55	0.41
**Pal2S1**	7428	4723	69	43	0.41	0.26	1.15	0.76	0.27	0.20
**Sty1S1**	4197	2220	32	17	0.13	0.12	0.37	0.35	0.11	0.12
**Sty2S1**	189	78	9	9	0.40	0.62	0.83	1.38	0.38	0.63
**Fav1A2**	2205	2507	63	39	0.68	0.82	1.90	2.21	0.46	0.60
**Fav2A2**	4298	2220	82	51	0.85	0.83	2.35	2.31	0.53	0.59
**Iso1M1**	5543	2663	43	22	0.23	0.03	0.59	0.11	0.16	0.04
**Iso2M1**	7881	4618	49	21	0.51	0.03	1.04	0.13	0.27	0.04
**Acr1S1**	8286	**5155**	33	14	0.07	0.02	0.22	0.08	0.06	0.03
**Acr2S1**	8092	4336	74	38	0.82	0.50	2.07	0.95	0.48	0.26
**Mon1S1**	4779	3055	54	35	0.42	0.55	1.16	1.27	0.29	0.36
**Mon2S1**	5276	3874	76	33	0.20	0.55	0.70	1.14	0.16	0.33
**Eup1S1**	7706	4426	64	39	0.69	0.78	1.57	1.82	0.38	0.50
**Eup2A2**	6630	3454	45	25	0.78	0.52	1.86	1.08	0.49	0.34
**Acr3S2**	2840	1415	38	25	0.65	0.66	1.73	1.43	0.48	0.44
**Acr4S2**	824	207	20	10	0.64	0.69	1.36	1.46	0.45	0.64
**Eup3S2**	1896	325	62	25	0.47	0.64	1.36	1.76	0.33	0.55
**Eup4S2**	1964	725	15	9	0.24	0.49	0.62	0.77	0.23	0.35
**Acr5S1**	2435	565	54	14	0.88	0.57	2.61	1.30	0.65	0.49
**Acr6S1**	3091	654	46	16	0.84	0.40	2.14	0.96	0.56	0.35
**Acr5D1**	2890	725	59	16	0.84	0.20	2.31	0.56	0.57	0.20
**Acr6D1**	2749	570	44	13	0.83	0.53	2.13	1.06	0.56	0.41
**Acr5A2**	2473	1585	43	16	0.39	0.38	0.83	0.88	0.22	0.32
**Acr6A2**	2609	455	45	11	0.84	0.41	2.11	0.90	0.56	0.37
**Acr5S2**	2705	419	40	8	0.46	0.51	1.13	1.10	0.31	0.53
**Acr6S2**	3090	527	38	12	0.52	0.48	1.21	1.05	0.33	0.42
**Sea1S1**	1288	860	**98**	71	**0.94**	**0.94**	**3.27**	3.24	0.71	0.76
**Sea2S2**	666	451	60	61	**0.94**	0.93	3.20	**3.31**	**0.78**	**0.81**
**Sea3S1**	2909	1916	88	**73**	0.81	0.88	2.34	2.77	0.52	0.65

The highest values in each column are in bold.

a N defined as the number of sequences.

b Operational taxonomic units (OTUs) formed at an evolutionary distance of <0.03 (or approximately97% similarity).

c Shannon index divided by the logarithm of the number of OTUs
