# Supplementary material for: A Newly Designed Primer Revealed High Phylogenetic Diversity of Endozoicomonas in Coral Reefs
Source: Microbes Environ. 2018 May 12;33(2):172–85. doi: 10.1264/jsme2.ME18054 (PMC6031392; doi:10.1264/jsme2.ME18054)
Supplement: Supplementary file 1 [file 33_172_s1.pdf]

Supplementary Information

Table S1. Locations and times of coral and seawater sample collection. Overall, 30 coral samples and 3 seawater samples were collected, with 19, 5 and 9 collected from Kenting, Hemei, and Kochi, respectively.

	2011			2012	
Sampling sites	March	Sept	Dec	April	Sept
**Kochi**		Acr5S1	Acr5D1	Acr5A2	Acr5S2
(32°46' 42.95"N,		Acr6S1	Acr6D1	Acr6A2	Acr6S2
132°43' 56.06"E)		Sea3S1			
**Kenting**	Iso1M1	Acr1S1		Fav1A2	
(21°56'58.3"N,	Iso2M1	Acr2S1		Fav2A2	
120°45'11.9"E)		Eup1S1		Eup2A2	
		Mon1S1			
		Mon2S1			
		Sty1S1			
		Sty2S1			
		Pal1S1			
		Pal2S1			
		Hel1S1			
		Hel2S1			
		Mil1S1			
		Mil2S1			
		Sea1S1			
**Hemei**					Acr3S2
(25°05'34.45"N,					Acr4S2
121°55'2.06"E)					Eup3S2
					Eup4S2
					Sea2S2

Abbreviations in sample names: For coral samples, Acr: *Acropora,* Iso: *Isopora,* Sty: *Stylophora,* Eup: *Euphyllia,* Mon: *Montipora,* Pal: *Palythoa,* Hel: *Heliopora,* Mil: *Millepora,* Fav: *Favia*. For seawater samples, Sea: seawater. For sampling time, M1: March 2011, S1: September 2011, D1: December 2011, A2: April 2012, S2: September 2012.

Table S2. Sequence information of partial COI region in coral. Similarity between sequences in samples with the same genus and blast result in GenBank.

Samples	Location	Similarity between sequences of samples within genus (%)	Blast result in NCBI	Identity (%)	Query cover (%)
**Iso1M1**	Kenting	100	*Isopora togianensis*	99	99
**Iso2M1**	Kenting		*Isopora togianensis*	99	99
**Mon1S1**	Kenting	99.9	*Montipora cactus*	99	99
**Mon2S1**	Kenting		*Montipora cactus*	98	99
**Sty1S1**	Kenting	100	*Stylophora pistillata*	99	99
**Sty2S1**	Kenting		*Stylophora pistillata*	99	99
**Pal1S1**	Kenting	100	*Palythoa tuberculosa*	100	92
**Pal2S1**	Kenting		*Palythoa tuberculosa*	100	92
**Hel1S1**	Kenting	100	*Heliopora coerulea*	99	99
**Hel2S1**	Kenting		*Heliopora coerulea*	99	99
**Mil1S1**	Kenting	100	*Millepora sp.*	90	99
**Mil2S1**	Kenting		*Millepora sp.*	90	99
**Fav1A2**	Kenting	100	*Favia rosaria*	99	97
**Fav2A2**	Kenting		*Favia rosaria*	99	97
**Eup1S1**	Kenting	99.1	*Euphyllia ancora*	99	98
**Eup2A2**	Kenting		*Euphyllia ancora*	98	98
**Eup3S2**	Hemei		*Euphyllia ancora*	99	98
**Eup4S2**	Hemei		*Euphyllia ancora*	99	98
**Acr1S1**	Kenting	100	*Acropora hyacinthus*	99	99
**Acr2S1**	Kenting		*Acropora hyacinthus*	99	99
**Acr3S2**	Hemei		*Acropora hyacinthus*	99	99
**Acr4S2**	Hemei		*Acropora hyacinthus*	99	99
**Acr5S1**	Kochi		*Acropora hyacinthus*	99	99
**Acr6S1**	Kochi		*Acropora hyacinthus*	99	99

Similarity of the mitochondrial COI genes among the same genus coral samples. To identify the genus of coral hosts, the similarity between COI genes of corals that belonged to the same genus were compared (Table S2). All COI sequences were blasted to the identified coral genera with high identity in GenBank, except the *Millepora* samples, which had the best hit with only 90% identity to *Millepora* sp. in the GenBank database. However, similarity between sequences from two *Millepora* samples was 100%. Most similarities were 100% between samples from the same coral genus, except *Euphyllia* and *Montipora* samples. Regarding similarity between *Euphyllia* samples, the variation was from COI sequences of Eup2A2 which had lower identity (98%) with sequences of *Euphyllia ancora* from GenBank. In contrast, *Acropora* samples had 100% similarity between their COI genes (regardless of where they were collected).

Table S3. Sequence information and diversity estimates after rarefying to an even 500 sequence depth.

Sample	N[a]		OTU[b]		Gini-Simpson		Shannon		Evenness[c]	
**Primer**	**V1V2**	**V3V4**	**V1V2**	**V3V4**	**V1V2**	**V3V4**	**V1V2**	**V3V4**	**V1V2**	**V3V4**
**Mil1S1**	497	498	2	4	0.10	0.02	0.21	0.07	0.30	0.05
**Mil2S1**	496	498	3	5	0.03	0.02	0.08	0.08	0.07	0.05
**Hel1S1**	497	497	8	4	0.09	0.03	0.25	0.09	0.12	0.06
**Hel2S1**	504	405	30	29	0.72	0.74	1.97	2.02	0.58	0.60
**Pal1S1**	493	496	29	17	0.85	0.72	2.32	1.51	0.69	0.53
**Pal2S1**	494	495	23	15	0.39	0.25	1.05	0.69	0.34	0.25
**Sty1S1**	496	498	7	8	0.11	0.11	0.30	0.32	0.15	0.15
**Sty2S1**	189*	78*	N.A.	N.A.	N.A.	N.A.	N.A.	N.A.	N.A.	N.A.
**Fav1A2**	493	499	32	23	0.67	0.82	1.81	2.17	0.52	0.69
**Fav2A2**	490	492	28	23	0.84	0.82	2.21	2.21	0.67	0.70
**Iso1M1**	495	497	9	4	0.22	0.02	0.51	0.06	0.23	0.04
**Iso2M1**	495	497	12	4	0.50	0.02	0.97	0.08	0.39	0.06
**Acr1S1**	495	499	4	3	0.06	0.02	0.15	0.06	0.11	0.06
**Acr2S1**	493	495	22	9	0.81	0.49	1.97	0.87	0.64	0.40
**Mon1S1**	498	496	18	15	0.42	0.55	1.10	1.20	0.38	0.44
**Mon2S1**	494	496	22	17	0.18	0.55	0.58	1.08	0.19	0.38
**Eup1S1**	495	496	15	14	0.68	0.78	1.49	1.75	0.55	0.67
**Eup2A2**	497	498	15	10	0.78	0.51	1.83	1.04	0.68	0.45
**Acr3S2**	499	498	26	16	0.64	0.66	1.71	1.39	0.52	0.50
**Acr4S2**	502	207*	20	N.A.	0.64	N.A.	1.37	N.A.	0.46	N.A.
**Eup3S2**	500	325*	37	N.A.	0.47	N.A.	1.33	N.A.	0.37	N.A.
**Eup4S2**	498	502	11	9	0.23	0.49	0.60	0.79	0.25	0.36
**Acr5S1**	499	501	33	14	0.88	0.57	2.57	1.31	0.73	0.50
**Acr6S1**	496	502	21	16	0.83	0.41	2.09	0.98	0.69	0.35
**Acr5D1**	499	502	33	16	0.84	0.21	2.28	0.57	0.65	0.21
**Acr6D1**	498	501	23	13	0.83	0.53	2.09	1.07	0.67	0.42
**Acr5A2**	495	497	13	11	0.38	0.38	0.75	0.85	0.29	0.36
**Acr6A2**	494	455	17	11	0.83	0.40	2.03	0.88	0.72	0.37
**Acr5S2**	496	419	19	8	0.45	0.51	1.07	1.11	0.36	0.53
**Acr6S2**	496	500	15	12	0.51	0.48	1.16	1.05	0.43	0.42
**Sea1S1**	512	451	**60**	61	**0.94**	0.93	**3.25**	**3.29**	**0.79**	**0.80**
**Sea2S2**	486	503	56	**71**	**0.94**	**0.94**	3.12	3.28	0.78	0.77

**Sea3S1**	492	495	37	52	0.80	0.88	2.23	2.72	0.62	0.69

The highest values in the number of OTUs, evenness, Shannon, and Gini-Simpson indices are in bold.

*The number of reads in samples are less than 400, and their diversity indices are presented as not available (N.A.)

[a]N defined as the number of sequences.

[b]Operational taxonomic units (OTUs) formed at an evolutionary distance of <0.03 (or about 97% similarity).

[c]Shannon index divided by the logarithm of the number of OTUs.

Table S4. The divergences within or between the *Endozoicomonas* and the outgroup sequences calculated from V1-V2 and V3-V4 region of 16S rRNA gene. Divergence of selected sequences was assessed by **distances** estimates of average evolutionary divergence with the Kimura 2-parameter model. All base positions containing gaps or missing data in the sequence alignment were discarded; thereafter, 305/415 informative sites in the alignment of V1-V2/V3-V4 region were available for analysis. Standard error of the mean distances was represented as S.E.

Divergence	Sequence resource	V1-V2		V3-V4	
		Distance	S.E.	Distance	S.E.
Within group	This study	0.046	0.006	0.053	0.007
	Reference	0.042	0.006	0.053	0.006
	Endozoicomonas	0.043	0.006	0.056	0.007
	Outgroup	0.122	0.013	0.124	0.013
Between groups	This study x Outgroup	0.102	0.011	0.127	0.013
	Reference x Outgroup	0.106	0.011	0.123	0.012
	Endozoicomonas x Outgroup	0.103	0.012	0.126	0.013
	This study x Reference	0.045	0.006	0.062	0.008

Table S5. Sequence information and diversity estimates, include singleton OTUs before rarefying, of *Endozoicomonas* community in coral and seawater samples from Kochi, as represented in the V1-V2 regions of the 16S rRNA gene, detected with bacterial universal primers (U) and *Endozoicomonas*-specific primers (S). The least number of sequences, except seawater sample from universal primer data, in all samples and data is 2496, marked by underline.

Sample	N[a]		OTU[b]		Richness[c]		Gini-Simpson		Shannon		Evenness[d]	
Primer	U	S	U	S	U	S	U	S	U	S	U	S
**Sea3S1**	1	2954	1	88	N.D.	0.416	N.D.	0.772	N.D.	2.17	N.D.	0.485
**Acr6S1**	12461	3330	138	69	0.46	0.529	0.865	0.842	2.35	2.15	0.476	0.507
**Acr5S1**	6647	2888	131	54	0.523	0.436	0.823	0.857	2.42	2.33	0.497	0.583
**Acr6D1**	8874	2895	126	56	0.433	0.509	0.817	0.827	2.29	2.11	0.474	0.525
**Acr5D1**	13066	3176	159	73	0.394	0.486	0.881	0.843	2.58	2.29	0.51	0.534
**Acr6A2**	6538	2766	99	60	0.42	0.492	0.798	0.84	2.16	2.13	0.469	0.52
**Acr5A2**	8078	2496	119	47	0.5	0.542	0.454	0.29	1.4	0.878	0.294	0.228
**Acr6S2**	7829	3118	88	58	0.393	0.492	0.462	0.395	1.4	1.13	0.312	0.279
**Acr5S2**	11467	2717	112	46	0.469	0.34	0.383	0.396	1.19	1.15	0.253	0.301

U=Bacterial universal primers; S=*Endozociomonas*-specific primers

N.D. = undetectable in sample.

[a]N defined as the number of sequences.

[b]Calculations were based on operational taxonomic units (OTUs) formed at an evolutionary distance of <0.03 (or about 97% similarity).

[c]Calculated as S / (N + 1) where S is the number of singleton OTUs and N is the total number of OTUs.

[d]Shannon index divided by the logarithm of the number of OTUs

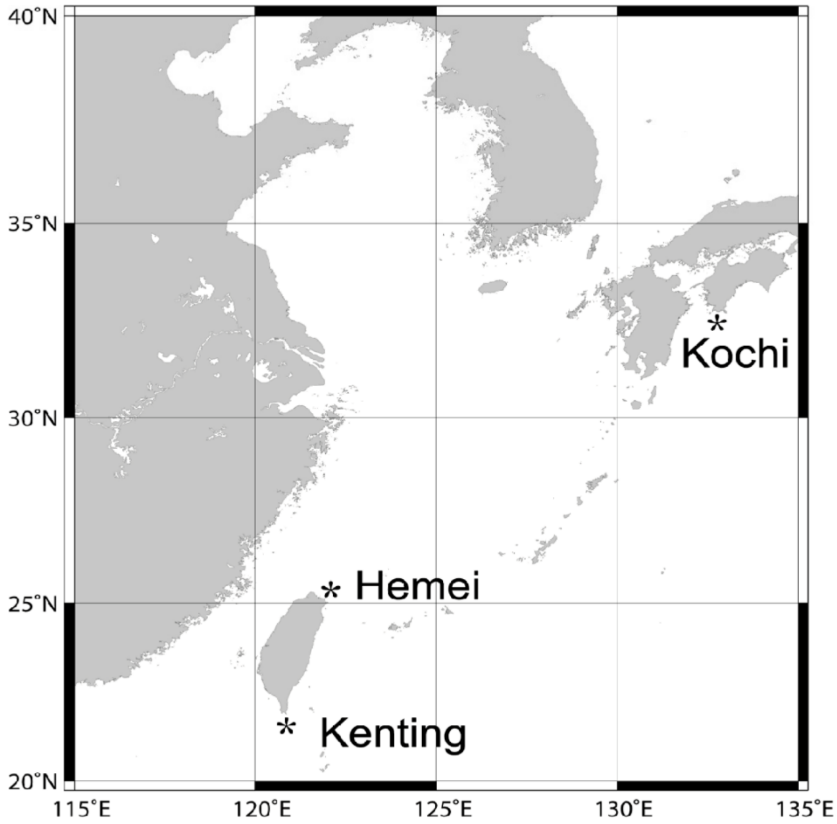

Fig S1. Locations for collection of corals and seawater samples. In total, 30 coral samples and 3 seawater samples were collected in March, September, and December 2011, and in April and September 2012. Nineteen were collected from tropical Kenting (21°56'58.3"N, 120°45'11.9"E), 5 from subtropical Hemei (25°05'34.45"N, 121°55'2.06"E), and 9 close to temperate Kochi (32°46' 42.95"N, 132°43' 56.06"E).

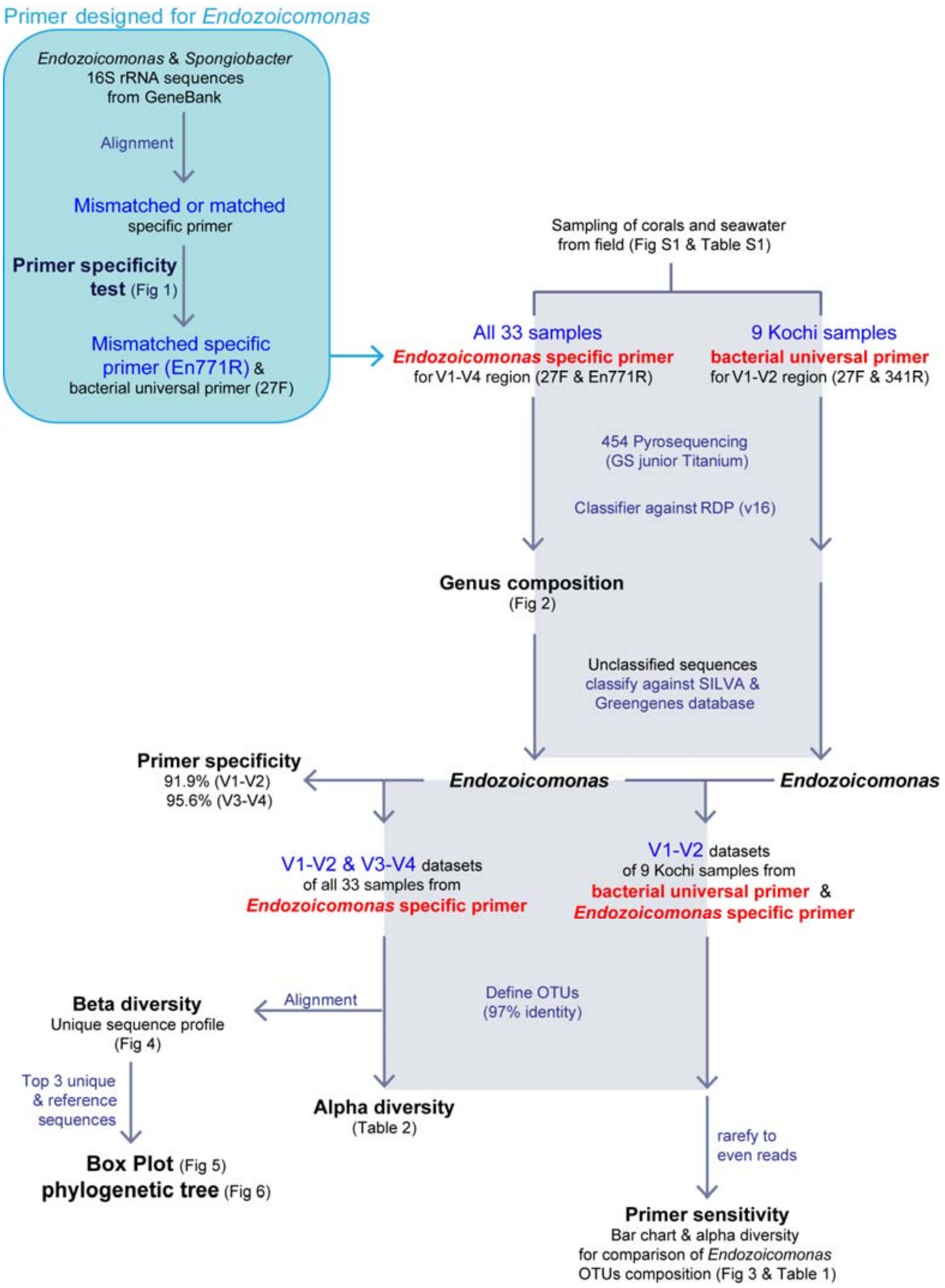

Fig S2. Flow chart for this study. Primer design and testing has a blue background, whereas steps in bold are also presented in other figures and tables.

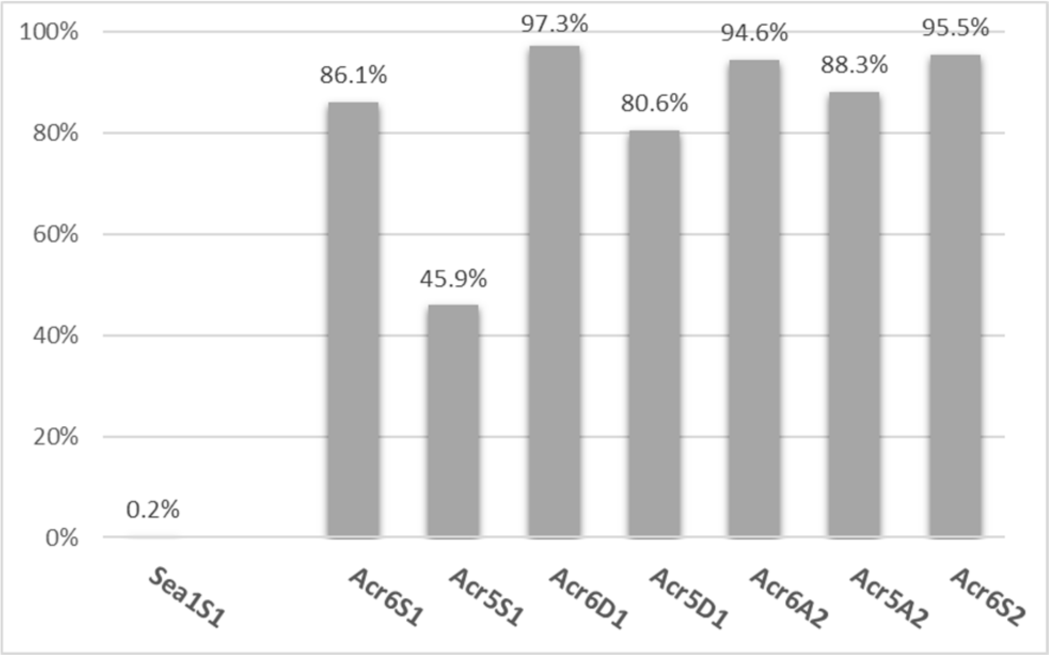

Fig S3. The relative abundance of *Endozoicomonas* in coral-associated bacterial community. The V1-V2 region of 16S rRNA gene were amplified using bacterial universal primer pair, 27F and 341R, in coral and seawater samples collected from Kochi. Only sequences assigned as *Endozoicomonas* were counted, and the relative abundance in all bacterial community of each sample were calculated. The Y axis is the relative abundance of *Endozoicomonas*, and X axis lists the sample name.

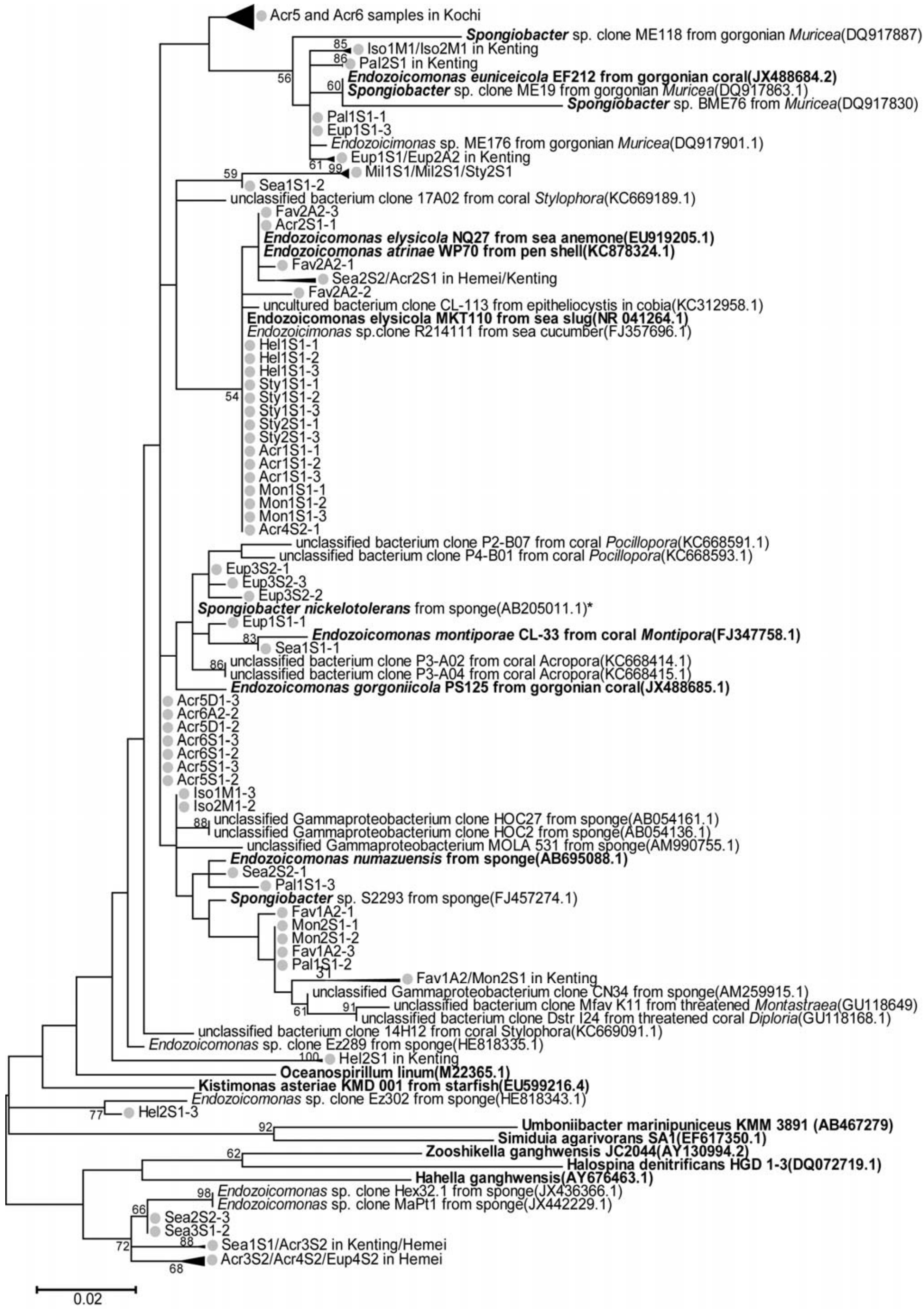
Acr5 and Acr6 samples in Kochi
Spongiobacter sp. clone ME118 from gorgonian Muricea(DQ917887)
85
Iso1M1/Iso2M1 in Kenting
86
Pal2S1 in Kenting
Endozoicomonas euniceicola EF212 from gorgonian coral(JX488684.2)
56
60
Spongiobacter sp. clone ME19 from gorgonian Muricea(DQ917863.1)
Spongiobacter sp. BME76 from Muricea(DQ917830)
Pal1S1-1
Eup1S1-3
Endozoicimonas sp. ME176 from gorgonian Muricea(DQ917901.1)
Eup1S1/Eup2A2 in Kenting
61
99
Mil1S1/Mil2S1/Sty2S1
59
Sea1S1-2
unclassified bacterium clone 17A02 from coral Stylophora(KC669189.1)
Fav2A2-3
Acr2S1-1
Endozoicomonas elysicola NQ27 from sea anemone(EU919205.1)
Endozoicomonas atrinae WP70 from pen shell(KC878324.1)
Fav2A2-1
Sea2S2/Acr2S1 in Hemei/Kenting
Fav2A2-2
uncultured bacterium clone CL-113 from epitheliocystis in cobia(KC312958.1)
Endozoicomonas elysicola MKT110 from sea slug(NR 041264.1)
Endozoicimonas sp.clone R214111 from sea cucumber(FJ357696.1)
Hel1S1-1
Hel1S1-2
Hel1S1-3
Sty1S1-1
54
Sty1S1-2
Sty1S1-3
Sty2S1-1
Sty2S1-3
Acr1S1-1
Acr1S1-2
Acr1S1-3
Mon1S1-1
Mon1S1-2
Mon1S1-3
Acr4S2-1
unclassified bacterium clone P2-B07 from coral Pocillopora(KC668591.1)
unclassified bacterium clone P4-B01 from coral Pocillopora(KC668593.1)
Eup3S2-1
Eup3S2-3
Eup3S2-2
Spongiobacter nickelotolerans from sponge(AB205011.1)*
Eup1S1-1
Endozoicomonas montiporae CL-33 from coral Montipora(FJ347758.1)
83
Sea1S1-1
86
unclassified bacterium clone P3-A02 from coral Acropora(KC668414.1)
unclassified bacterium clone P3-A04 from coral Acropora(KC668415.1)
Endozoicomonas gorgoniicola PS125 from gorgonian coral(JX488685.1)
Acr5D1-3
Acr6A2-2
Acr5D1-2
Acr6S1-3
Acr6S1-2
Acr5S1-3
Acr5S1-2
Iso1M1-3
Iso2M1-2
88
unclassified Gammaproteobacterium clone HOC27 from sponge(AB054161.1)
unclassified Gammaproteobacterium clone HOC2 from sponge(AB054136.1)
unclassified Gammaproteobacterium MOLA 531 from sponge(AM990755.1)
Endozoicomonas numazuensis from sponge(AB695088.1)
Sea2S2-1
Pal1S1-3
Spongiobacter sp. S2293 from sponge(FJ457274.1)
Fav1A2-1
Mon2S1-1
Mon2S1-2
Fav1A2-3
Pal1S1-2
31
Fav1A2/Mon2S1 in Kenting
unclassified Gammaproteobacterium clone CN34 from sponge(AM259915.1)
91
unclassified bacterium clone Mfav K11 from threatened Montastraea(GU118649)
61
unclassified bacterium clone Dstr I24 from threatened coral Diploria(GU118168.1)
unclassified bacterium clone 14H12 from coral Stylophora(KC669091.1)
Endozoicomonas sp. clone Ez289 from sponge(HE818335.1)
100
Hel2S1 in Kenting
Oceanospirillum linum(M22365.1)
Kistimonas asteriae KMD 001 from starfish(EU599216.4)
Endozoicomonas sp. clone Ez302 from sponge(HE818343.1)
77
Hel2S1-3
92
Umboniibacter marinipuniceus KMM 3891 (AB467279)
Simiduia agarivorans SA1(EF617350.1)
62
Zooshikella ganghwensis JC2044(AY130994.2)
Halospina denitrificans HGD 1-3(DQ072719.1)
Hahella ganghwensis(AY676463.1)
98
Endozoicomonas sp. clone Hex32.1 from sponge(JX436366.1)
Endozoicomonas sp. clone MaPt1 from sponge(JX442229.1)
66
Sea2S2-3
Sea3S1-2
88
72
Sea1S1/Acr3S2 in Kenting/Hemei
Acr3S2/Acr4S2/Eup4S2 in Hemei
68
0.02

Fig S4. Phylogenetic tree of *Endozoicomonas* and *Spongiobacter* V1-V2 region in 16S rRNA sequences and closely related bacteria. Phylogeny was constructed with the top 3 abundant unique sequences of V1-V2 region in 16S ribosomal RNA gene in each sample, and other members of the *Endozoicomonas*, *Spongiobacter* and outgroup representative sequences in the family, *Hahellaceae*, the order, *Oceanospirillales,* and the phylum, *Gamma-proteobacteria*, in GenBank. Sequences collected from this study were marked as grey circles. Numbers shown on branches are bootstrap values (1000 bootstraps; those < 50% are not shown). The scale bar corresponds to 0.02 substitutions per nucleotide position. Bold font denotes *Spongiobacter* or isolated strains of *Endozoicomonas*, and the first *Spongiobacter* sequence proposed in 2005 was marked with an asterisk. The ourgroup representative sequences cannot be separated well from *Endozoicomonas* sequences in this phylogenetic tree.

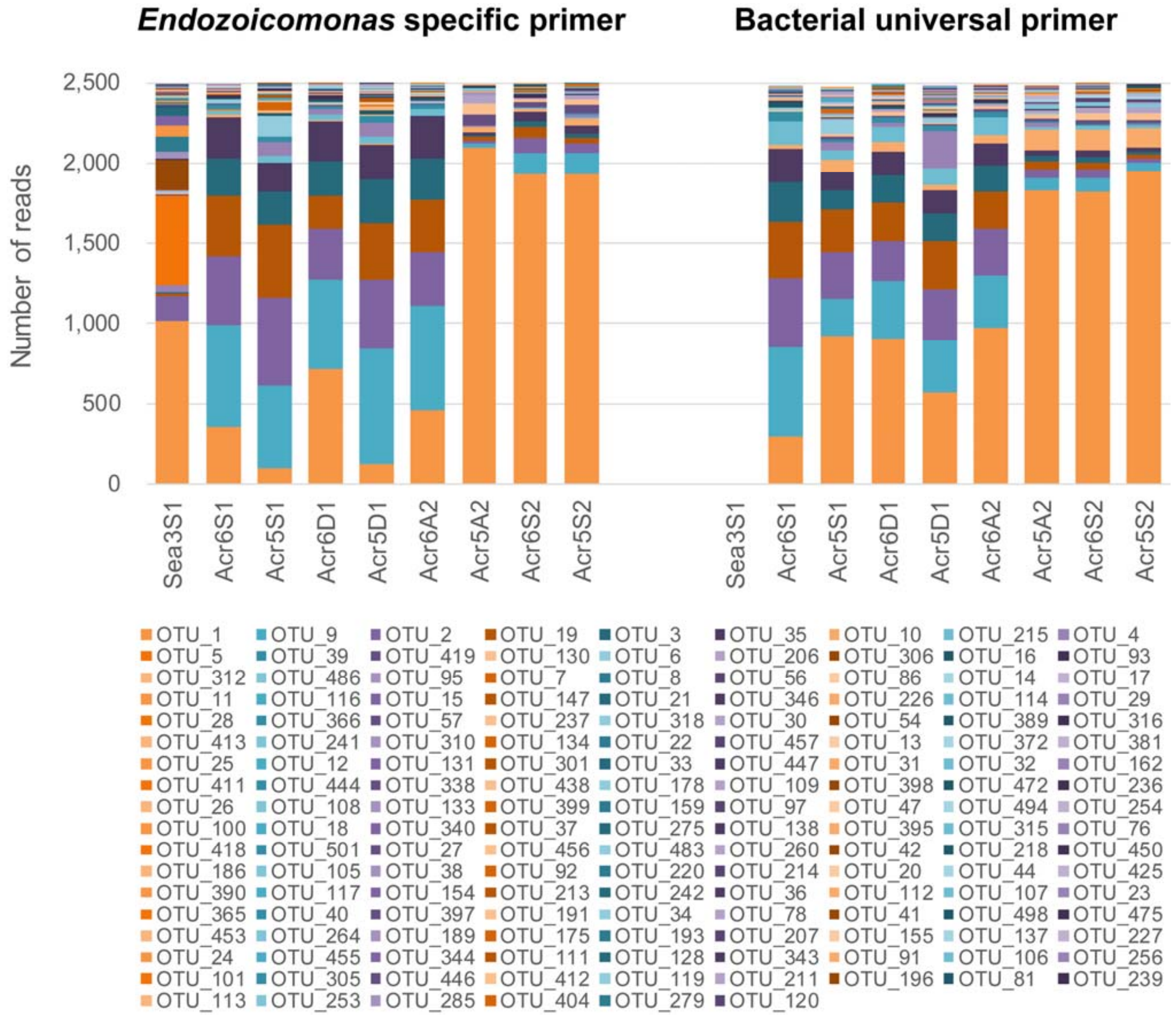

Figure S5. Bar chart of *Endozoicomonas* OTUs composition in coral and seawater samples collected from Kochi with color keys for all OTUS in Figure 3. After rarefying and excluding singleton OTUs, there were 138 and 103 *Endozoicomonas* OTUs in the specific primer and universal primer datasets, respectively. In total, there were 159 OTUs from all samples and 82 OTUs can be found in both datasets from *Endozoicomonas* and universal primers. The order of OTUs are listed from the highest to the lowest relative abundances in all samples.
